# Variation in admission rates between psychiatrists on call in a university teaching hospital

**DOI:** 10.1186/s12991-018-0199-x

**Published:** 2018-07-10

**Authors:** Jay Moss, Dippy Nauranga, Doyoung Kim, Michael Rosen, Karen Wang, Krista Lanctot

**Affiliations:** 1 Department of Psychiatry, Sunnybrook Health Sciences Centre, Toronto, Canada; 2 Department of Psychiatry and Neuropsychopharmacology Research Program, Sunnybrook Health Sciences Centre, Toronto, Canada

## Abstract

**Background:**

Hospital-based physicians must routinely decide whether patients receiving care in the emergency room require admission to an acute care bed. We endeavoured to understand clinician-related factors that influence the decision to admit.

**Methods:**

We retrospectively examined data collected between August 1, 2013 and July 31, 2015 for patients triaged as mental health assessments in the emergency department of a university teaching hospital. We identified 1530 unique cases who had been reviewed by the staff psychiatrist for a decision on whether to admit to an acute care bed. Patient and physician characteristics were analyzed by standard descriptive methods, comparative statistics (Chi square and analysis of variance) and regression analyses using SPSS version 24.0 (IBM Corp. Armonk, NY, USA).

**Results:**

There were no differences in patient characteristics in the clinical encounters reviewed by different staff psychiatrists. The physician factor found significant in deciding whether to admit the patient was assignment to PES (psychiatric emergency services). This appeared to be the only physician variable impacting the decision to admit a patient with PES psychiatrists admitting less often than their colleagues (*p* = 0.018, Table 3). The effect size of the variable in terms of odds ratio was 0.592.

**Interpretation:**

Training and practice in emergency psychiatry lead to lower admission rates when these clinicians are on call. Training in emergency psychiatry for all psychiatrists participating in a call pool may result in lowered admission rates.

## Background

The decision to admit a patient from a teaching hospital emergency department is generally at the discretion of the consultant specialist following a referral from the emergency room (ER) physician. The patient is usually first assessed by the specialty resident on call before reviewing the patient with the specialty consultant on call. This arrangement is fairly standard across North American teaching hospitals with some variation at different locations. For the most part, the decision to admit a patient to hospital is at the discretion of the specialist consultant with input from the resident.

Previous studies looking at admissions to psychiatry have examined patient factors that tend to predict admission. These factors include, but are not limited to, elevated suicide risk, specific diagnosis (schizophrenia and affective disorders) and a history of poor impulse control reflected in suicide attempts, self-harm behaviours, and substance abuse [1, 2]. External and service factors that may influence disposition such as timeliness of access to community-based treatments also influence the decision to admit [3]. There have also been efforts to study the decision-making process that determines whether a patient is admitted to psychiatry.

The psychiatric emergency service (PES) has evolved over the past 25 years as a dedicated program embedded in the emergency rooms of many North American teaching hospitals. The psychiatry resident training program at the University of Toronto requires every second year psychiatry resident to complete a 5 weeks rotation on a PES. The rotation combines supervised clinical encounters with PES staff i n addition to didactic and small group l earning in emergency psychiatry. Psychiatry residents are required to participate in on-call duties over the course of their 5 years residency. This training model has been evaluated by resident performance in clinical and examination settings as well as the acquisition of core competencies considered essential in the emergency psychiatry setting.

There is general consensus that the decision-making process is complex with variability between psychiatrists depending on what clinical criteria are applied in arriving at the decision to admit [4, 5]. There have been few studies examining inter-rater reliability between PES providers and disposition decisions. Consensus between clinicians on the decision to admit was found to be poor [6]. One possible factor that may contribute to this variability is clinician experience. In support of this, an educational program offered to second year psychiatry residents was effective in reducing admission to the level of more senior residents [7]. However, other factors contributing to the decision to admit have not been elucidated.

We performed a retrospective review of admissions to psychiatry by on-call staff at a large university affiliated general hospital to determine whether there were differences in rates of admission between staff psychiatrists with the goal of identifying factors associated with these differences.

## Setting

Sunnybrook Health Sciences Centre is a large teaching hospital affiliated with the University of Toronto located in Toronto, Ontario, Canada with identified priority programs in a number of clinical areas including Brain Sciences. The emergency department provides triage and clinical assessment by the ER physician for all patients. The ER physician decides whether to refer the patient to the PES for urgent assessment. During weekday hours (8:30 a.m. to 4:30 p.m.), there is a dedicated PES team comprised of an RN, psychiatry resident, and staff psychiatrist. Outside of these hours, the PES RN is available until 11:00 p.m. with the on-call psychiatry resident and staff psychiatrist (most of whom do not work in PES) providing overnight and weekend coverage. The ER physician may refer to the PES RN for advice on management and disposition. Alternatively, the ER physician may refer the patient to the psychiatry resident on call who will assess the patient and then review (usually by phone) with the psychiatry staff on call who then decides whether the patient is admitted to psychiatry. All departmental staff psychiatrists working greater or equal to 0.6 full-time equivalents are required to participate in call. Of the 32 staff psychiatrists participating in the call pool, 4 were members of the PES team and shared weekday coverage allocated by a dedicated schedule.

## Method

We retrospectively collected data from the period between August 1, 2013 and July 31, 2015. During this period, there were a total of 115,050 patient visits registered in the emergency department. From this total, 6428 (5.6%) were triaged as “Mental Health Assessment”.

Following an examination by the ER physician, 778 (12%) of these patients were referred to the PES RN who met with the patient and reviewed their findings and recommendations with the ER physician before discharge. The ER physicians referred 1574 (25%) for a consultation with the on-call psychiatry resident. The patients seen by the psychiatry resident were either admitted to psychiatry or discharged. In general, the ER physician would refer high risk and complex patients to the on-call psychiatry resident. At the time of referral, the patient may be voluntary or detained under the Mental Health Act (MHA) of Ontario for a psychiatric assessment. Voluntary patients may choose to leave the hospital before the psychiatric assessment is completed. Upon completion of the assessment, patient disposition options include discharge from the ER (often with outpatient follow-up), admission to the acute psychiatry inpatient unit as a voluntary patient or admission as an involuntary patient. The focus for this study w as the binary outcome of admission or non-admission and we did not account for voluntary or involuntary status. Out of the 1574 referrals, encounters were excluded if there was no staff psychiatrist identified, the patient was reviewed by a staff psychiatrist who saw a very minimal number of patients (defined as < 8 patients during the study season), or the encounter resulted in an admission other than the psychiatric inpatient unit such as a surgical unit or the critical care unit (CCU). All other encounters were included in the database.

Towards the end of the study period, a survey of call experience was circulated to all psychiatrists participating in the call pool. Each psychiatrist received a survey the day following call and was asked to consider their most recent call experience. The survey enlisted a Likert scale to quantify subjective perceptions of confidence levels and opportunities for learning while on call. Anchor points ranged from 1 (strongly disagree) to 5 (strongly agree). Twenty-eight of 31 (90%) psychiatrists completed the survey.

### Statistical analysis

Analysis of data was completed by standard descriptive methods, comparative statistics (Chi square and analysis of variance, ANOVA) and regression analyses using SPSS version 24.0 (IBM Corp. Armonk, NY, USA). Patient and clinician factors in those admitted versus not were compared using the Pearson Chi squared test for categorical variables and ANOVA for continuous variables to identify factors significantly associated with admission to psychiatry.

Patient factors collected included sex, age, age category [youth (ages 14–21 inclusive), general or geriatric (65 and older)], multiple visits during the study timeframe and previous admission experience within the study timeframe. Patient diagnosis categories were based on International Classification of Disease (ICD-10-CM) and included dementia, delirium, substance-related disorders, schizophrenic and other psychotic disorders, bipolar and related disorders, depression, anxiety, obsessive compulsive and related disorders, trauma and stress related disorders, dissociative disorders, somatic disorders, eating disorders, personality disorders, neurodevelopmental disorders and other miscellaneous diagnoses. Clinician characteristics observed were affiliation with age related division (youth, geriatric, or general), gender, practice in a specialty (psycho-oncology, mood and anxiety disorders, consultation liaison, psychiatric emergency services, and women’s mental health) versus general psychiatry, years of practice, and primary type of care practice (inpatient or outpatient). Another Pearson Chi squared test was performed to compare the disposition of the groups of those with specialty match (i.e., youth patients seen by youth specialties, and geriatric patients by geriatric specialties) and those who were not matched.

Informed by the comparative statistics, we looked at the four significantly different physician factors plus two factors from the survey results (perceived competency while on-call survey scores [1 strongly disagree to 5 strongly agree] and opportunities for learning, while on-call survey scores [1 strongly disagree to 5 strongly agree]) completed by the physicians to assess their impact on admissions while controlling for significant patient factors determined from the previous comparative statistics. This was completed using multivariate logistic regression (Enter method), following the multicollinearity test of all variables. 95% confidence interval (95% Cl), and *p* value were reported for regression analyses.

## Results

Of the 1574 referrals for a psychiatry consultation with the on-call resident, 1530 cases met inclusion criteria. In total, 31 physicians saw these encounters with a mean of 49.4 encounters per physician (range 13–103 encounters per physician). Physician and patient characteristics for the 1530 encounters are shown in Table 1. Patients were more commonly female (61.2%) with a mean age of 37.3 ± 18.6 years. In addition, the emergency visits were mostly composed of general age groups (64.7%). Physicians were typically male (71.3%) with a mean of 14.3 ± 8.7 years of practice and typically practiced outpatient care (74.4%). A small proportion of the visits were specialty-matched (8.2%).

**Table 1:** Clinician and patient factors

	Total *n* = 1530
Patient factors	
Male (%)	593 (38.8)
Age (years ± SD)	37.3 ± 18.6
Youth (14–21) (%)	400 (26.1)
General (22–64) (%)	990 (64.7)
Geriatrics (65+) (%)	136 (8.9)
Others (< 14) (%)	4 (0.3)
Diagnoses (ICD-10-CM category)	
Dementia and delirium (%)	21 (1.4)
Substance-related disorders (%)	99 (6.5)
Schizophrenia and other psychotic disorders (%)	241 (15.8)
Bipolar and related disorders (%)	129 (8.4)
Depressive disorders (%)	350 (22.9)
Anxiety disorders (%)	107 (7.0)
Obsessive compulsive and related disorders (%)	12 (0.8)
Trauma and stress-related disorders (%)	71 (4.6)
Dissociative disorders (%)	3 (0.2)
Somatic symptom and related disorders (%)	3 (0.2)
Eating disorders (%)	1 (0.1)
Personality disorders (%)	27 (1.8)
Neurodevelopmental disorders (%)	1 (0.1)
Miscellaneous diagnoses (%)	465 (30.4)
Multiple visits	449 (29.3)
With previous admission (%)	247 (16.1)
Specialty match (%)	125 (8.2)

	Total *n* = 1530
Physician factors	
Male (%)	1091 (71.3)
Years of practice (mean ± SD)	14.3 ± 8.7
PES training (%)	308 (20.1)
Specialist/highly specialized (%)	247 (16.1)
Sub-division of primary specialty area (%)	
Youth	395 (25.8)
Geriatric	143 (9.3)
General	753 (49.2)
PES	308 (20.1)
Inpatient	240 (15.7)
Outpatient	166 (10.8)
Consultation liaison	39 (2.5)
Women’s Mental Health	91 (5.9)
Oncology	48 (3.1)
OCD	43 (2.8)
CBT	42 (2.7)
Neuropsych	15 (1.0)
Other	239 (15.6)
Frequent care practice type (%)	
Outpatient	1139 (74.4)
Inpatient	391 (25.6)

Comparative statistics resulting in patients admitted versus not are shown in Table 2. Patient factors that differed significantly between the groups were patient age, geriatric patients, previous admission, and those with diagnosis of dementia, SCZ, BPD, anxiety, trauma/stress, and other MISC disorders. The relatively low number of geriatric patients (8.9%) is likely due to a number of factors. There is a well-established community-based psychogeriatric program at Sunnybrook that provides long-term follow-up and likely reduces the hospitalization rate for this population. Geriatric patients in the Sunnybook ER who present with Delirium a referred to General Internal Medicine with psychiatry providing a consultative role. Geriatric patients with Dementia are referred to the social work program for optimization of community resources as well as rapid access to long-term care placement. Patients with Dementia in the ER are also seen by Geriatric Psychiatry in a consultative role to optimize medication management. Additional patient characteristics such as income, housing status, cultural identity, and other demographic factors were not included in this study as the focus was on clinician variables influencing the decision to admit. There were no differences in patient characteristics in the clinical encounters reviewed by staff psychiatrists. Significantly different clinician factors were clinicians’ gender, PES training status, practice in specialty or general, and those with the specialty in Psycho-Oncology, and Inpatient Care.

**Table 2:** Admission rates for each factor

	Number admitted (*n* = 780)	Percent admittance (%)	*x*^2^ or *F*(*df*)	*p* value
Clinician factors				
Gender				
Female	243	55.4	4.7 (1)	0.030
Male	537	49.2		
Years of practice			2.3 (1)	0.126
PES training	126	40.9	15.7 (1)	< 0.0005
Specialist/highly specialized	153	61.9	14.2 (1)	< 0.0005
Specialty area				
Youth	192	48.6	1.2 (1)	0.273
Geriatric	75	52.4	0.1 (1)	0.712
Inpatient	140	58.3	6.2 (1)	0.013
Outpatient	81	48.8	0.4 (1)	0.551
Consultation liaison	20	51.3	0.0 (1)	0.970
Women’s mental health	52	57.1	1.5 (1)	0.225
Psycho-oncology	34	70.8	7.8 (1)	0.005
OCD	24	55.8	0.4 (1)	0.520
CBT	26	61.9	2.1 (1)	0.151
Neuropsych	10	66.7	1.5 (1)	0.222
Frequent care practice type among all physicians				
Outpatient	566	49.7	3.0 (1)	0.085
Inpatient	214	54.7		
Patient factors				
Gender				
Female	484	51.5	0.3 (1)	0.577
Male	297	50.1		
Age			6.3 (1)	0.012
Age groups				
Youth (14–21)	193	48.3	1.6 (1)	0.204
General (22–64)	501	50.6	0.2 (1)	0.692
Geriatrics (65+)	85	62.5	7.9 (1)	0.005
Diagnoses (ICD-10-CM category)				
Dementia and delirium	16	76.2	5.4 (1)	0.020
Substance-related disorders	44	44.4	1.8 (1)	0.179
Schizophrenia and other psychotic disorders	179	74.3	62.1 (1)	< 0.’0005
Bipolar and related disorders	94	72.9	27.0 (1)	< 0.0005
Depressive disorders	168	48.0	1.6(1)	0.204
Anxiety disorders	21	19.6	45.3 (1)	< 0.”0005
Obsessive compulsive and related disorders	5	41.7	0.4 (1)	0.517
Trauma and stress-related disorders	19	26.8	17.5 (1)	< 0.0005
Dissociative disorders	2	66.7	0.3 (1)	0.586
Somatic symptom and related disorders	0	0.0	3.1 (1)	0.077
Eating disorders	0	0.0	1.0 (1)	0.308
Personality disorders	15	55.6	0.2 (1)	0.631
Neurodevelopmental disorders	1	100	1.0 (1)	0.327
Miscellaneous diagnoses	216	46.5	5.5 (1)	0.019
Multiple visits	244	54.3	2.9 (1)	0.090
Previous admission	150	60.7	11.2 (1)	0.001
Specialty match				
Yes	81	51.3	0.0 (1)	0.940
No	699	50.9		

Multivariate regression analysis examined the four physician factors (years of practice in psychiatry, practice in general or specialty, practice in PES or other, specialty in outpatient or inpatient), which were determined to be significantly different between the groups of the patients admitted and not, plus the two survey results (subjective ratings on competency and learned-something-new surveys) involved in the decision to admit the patient, adjusted by significantly different patient factors shown in comparative statistics analysis (Table 2). Prior to multivariate logistic regression, all variables in the model passed the multicollinearity test (all above the tolerance level of 0.4).

Patient and environmental factors associated with a greater likelihood of admission include previous admissions (Exp(*B*) = 1.64, *p* = 0.002), geriatric patients (Exp(*B*) = 1.69, *p* = 0.016), diagnosis of schizophrenia (Exp(*B*) = 3.41, *p* < 0.0005), bipolar disorder (Exp(*B*) = 2.86, *p* < 0.0005), or related disorders. On the other hand, patients with a diagnosis of anxiety, trauma or stress related disorders were less likely to be admitted (Exp(*B*) = 0.28, *p* < 0.0005; Exp(*B*) = 0.46, *p* = 0.009, respectively, Table 3).

**Table 3:** Multivariate logistic regression analysis

	Exp(*B*)	95% CI for Exp(*B*)		*df*	*p* value
		Lower	Upper		
Patient factors					
Previous admittance	1.639	1.200	2.238	1	0.002
Geriatric group of patients	1.688	1.104	2.582	1	0.016
Dementia and delirium	2.568	0.873	7.558	1	0.087
Schizophrenia or psychotic disorders	3.414	2.373	4.911	1	< 0.0005
Bipolar or related disorders	2.859	1.818	4.494	1	< 0.0005
Anxiety disorder	0.283	0.166	0.482	1	< 0.0005
Stress or trauma	0.459	0.255	0.824	1	0.009
Miscellaneous diagnoses	1.022	0.778	1.343	1	0.876
Psychiatrist factors					
Years of practice	0.992	0.978	1.006	1	0.268
Learned _Something_new survey score of 2 relative to score of 1	1.078	0.500	2.324	1	0.848
Learned _Something_new survey score of 3 relative to score of 1	0.955	0.556	1.641	1	0.868
Learned _Something_new survey score of 4 relative to score of 1	1.027	0.693	1.523	1	0.893
Learned _Something_new survey score of 5 relative to score of 1	1.125	0.708	1.788	1	0.619
Competency survey score of 4 relative to score of 3	1.075	0.641	1.805	1	0.784
Competency survey score of 5 relative to score of 3	0.923	0.540	1.577	1	0.769
Specialty in inpatient versus outpatient	1.061	0.746	1.509	1	0.743
Practice in specialty versus general	0.890	0.642	1.233	1	0.484
Specialty in PES versus other	0.592	0.384	0.913	1	0.018

The physician factor found significant in deciding whether to admit the patient was assignment to PES. This appeared to be the only physician variable impacting the decision to admit a patient with PES psychiatrists admitting less often than their colleagues (*p* = 0.018, Table 3). The effect size of the variable in terms of odds ratio (OR) was 0.592.

A subanalysis within the PES psychiatrists examined whether greater clinical coverage had an impact on admission rates. Within the PES staff complement, weekday coverage was stratified with one psychiatrist providing 40% of the weekday coverage, one providing 30%, one providing 20% and one providing 10% of the weekday coverage. The differing amount of coverage by PES psychiatrists did not impact the proportion of patients admitted (*X*^2^ = 2.75, *df* = 3, *p* = 0.432).

## Discussion

Previous studies have focused on patient and systems factors that influence the decision to admit a patient seen in the emergency department to the psychiatry inpatient unit [8]. There has been some consideration of clinician factors that influence the decision to admit but little is known about these variables [9]. In addition to clinician variables, efforts to integrate input from patients and their families add additional variables into an already complex process of decision making [10].

We reviewed consecutive patient encounters in the emergency department of a large teaching hospital to measure and understand differences in the admission rates between on-call psychiatrists. Before the decision to admit, the patient would have been seen by a succession of clinicians including the ER triage RN, ER physician, and the psychiatry resident on call. In addition, collateral information may have been obtained from a family member or other person known to the patient. Following a telephone review and discussion between the psychiatry resident and staff psychiatrist on call, the staff psychiatrist would ultimately decide whether to admit the patient.

Psychiatrists who spend a portion of their clinical duties in PES were less likely to admit. In this study, there was a compliment of 4 psychiatrists providing PES coverage during weekday hours (8:30 to 4:30) from a total complement of 32 staff. After hours, call (weeknights and weekends) was shared equally between all staff psychiatrists including those working in PES.

These results appear both predictable and surprising. Psychiatrists working in the specialized area of PES would have more experience with the patient population that attends care in the emergency department. This may lead to a greater risk tolerance for discharging a patient with a severe mental illness. However, psychiatrists working in an inpatient setting would also be familiar with this population. Inpatient psychiatrists may apply an inherent bias towards admission based on their observations of the beneficial effects of admission and possibly to reinforce the value of their work. There may also be a financial incentive to admit patients if beds are not optimally utilized.

Challenging the variation in admission rates is the fact that all staff psychiatrists had undergone resident training in emergency psychiatry and would have logged many hours on call during residency with exposure to the same population they would be overseeing as a staff psychiatrist. These skills and practices should be enduring.

In the model of clinical care provided in a university teaching hospital, the interface between psychiatry resident and staff may also be worth examining to determine relative influences. Ultimately, it is the staff psychiatrist who decides whether to admit but this decision may be influenced by the experience and attitude of the resident providing the information. Given similarities in training and workplace cultures, it is not surprising that concordance in diagnosis between psychiatric residents and staff is strong [11]. A more complex dynamic is the unique relationship between resident and staff psychiatrist. In addition to the “power” imbalance, there may be subtle relational factors that influence outcome. For instance, a confident and outspoken resident may have a significant influence on the staff psychiatrist. Our study did not examine relational factors between residents and staff psychiatrists and how they may have influenced decision making.

This study did not evaluate outcomes of the decision to admit. Nearly, one-third of the study population had multiple visits to the ER during the study period. Slightly more than half (54%) of these patients were admitted. Although reasons for admission were not evaluated, multiple visits may represent a failure to meet patient needs. This subpopulation warrants further study. On the surface, lower admission rates may appear preferable with respect to inpatient bed utilization, but may not result in better patient outcomes. It is possible that patients who were not admitted suffered worse outcomes ranging from eventual admission to another facility to catastrophically, suicide. Future research must incorporate meaningful outcome measures to determine whether the decision to admit or discharge was the “right” decision.

If lower admission rates are considered a preferred outcome, these results may be instructive. Educational programs offered by PES staff to the staff psychiatrist call pool may impact outcomes. This educational model was shown to be effective in reducing admission rates for junior psychiatry residents [7]. Perhaps, all staff psychiatrists participating in the call pool should take regular weekday shifts in PES to increase exposure to this decision-making practice. Of course, this would expand the PES staff psychiatrist complement and dilute the experience resulting in an overall increase in admission rates.

Although this study examined admissions to psychiatry, the results may be generalizable to other specialties such as general internal medicine, where a subset of the call pool has additional experience in consultations to the emergency department.

Considering the value of inpatient beds in our health care systems, it is important to examine potentially modifiable factors influencing the decision to admit as well as patient outcomes related to these decisions.
